# Host–Virus Cophylogenetic Trajectories: Investigating Molecular Relationships between Coronaviruses and Bat Hosts

**DOI:** 10.3390/v16071133

**Published:** 2024-07-15

**Authors:** Wanlin Li, Nadia Tahiri

**Affiliations:** Department of Computer Science, University of Sherbrooke, 2500 Bd University, Sherbrooke, QC J1K 2R1, Canada; wanlin.li@usherbrooke.ca

**Keywords:** bats, cophylogeny, coronaviruses, horizontal gene transfer, phylogeny

## Abstract

Bats, with their virus tolerance, social behaviors, and mobility, are reservoirs for emerging viruses, including coronaviruses (CoVs) known for genetic flexibility. Studying the cophylogenetic link between bats and CoVs provides vital insights into transmission dynamics and host adaptation. Prior research has yielded valuable insights into phenomena such as host switching, cospeciation, and other dynamics concerning the interaction between CoVs and bats. Nonetheless, a distinct gap exists in the current literature concerning a comparative cophylogenetic analysis focused on elucidating the contributions of sequence fragments to the co-evolution between hosts and viruses. In this study, we analyzed the cophylogenetic patterns of 69 host–virus connections. Among the 69 host–virus links examined, 47 showed significant cophylogeny based on ParaFit and PACo analyses, affirming strong associations. Focusing on two proteins, ORF1ab and spike, we conducted a comparative analysis of host and CoV phylogenies. For ORF1ab, the specific window ranged in multiple sequence alignment (positions 520–680, 770–870, 2930–3070, and 4910–5080) exhibited the lowest Robinson–Foulds (RF) distance (i.e., 84.62%), emphasizing its higher contribution in the cophylogenetic association. Similarly, within the spike region, distinct window ranges (positions 0–140, 60–180, 100–410, 360–550, and 630–730) displayed the lowest RF distance at 88.46%. Our analysis identified six recombination regions within ORF1ab (positions 360–1390, 550–1610, 680–1680, 700–1710, 2060–3090, and 2130–3250), and four within the spike protein (positions 10–510, 50–560, 170–710, and 230–730). The convergence of minimal RF distance regions with combination regions robustly affirms the pivotal role of recombination in viral adaptation to host selection pressures. Furthermore, horizontal gene transfer reveals prominent instances of partial gene transfer events, occurring not only among variants within the same host species but also crossing host species boundaries. This suggests a more intricate pattern of genetic exchange. By employing a multifaceted approach, our comprehensive strategy offers a nuanced understanding of the intricate interactions that govern the co-evolutionary dynamics between bat hosts and CoVs. This deeper insight enhances our comprehension of viral evolution and adaptation mechanisms, shedding light on the broader dynamics that propel viral diversity.

## 1. Introduction

In recent years, bats have garnered substantial attention within the scientific community due to their pivotal role as reservoirs for emerging viruses [1,2]. Their significance is underscored by their extensive geographical distribution, encompassing a diverse array of around 1400 species spanning 21 families and over 200 genera [3]. Furthermore, bats possess distinct ecological and physiological characteristics that make them capable hosts for a wide spectrum of viruses. For instance, their remarkable trait of vagility sets them apart from other mammals, facilitating their engagement in long–distance dispersal. Moreover, their tendency to form large, colonial populations further enhances their potential as reservoirs for diverse emerging viral pathogens [4]. These unique and remarkable attributes exhibited by bats have led to their unequivocal recognition as crucial candidates for harboring and transmitting novel, potentially pathogenic viruses.

Among these viruses, coronaviruses (CoVs) stand out as a prominent group that has garnered considerable attention. Classified within the expansive order *Nidovirales*, suborder *Cornidovirineae*, and family *Coronaviridae*, CoVs form one of the most extensive families of viruses [5]. The notable diversity of CoVs can be attributed to three key factors that significantly influence their genetic flexibility and adaptability. Firstly, the RNA–dependent RNA polymerase of CoVs displays a remarkable degree of infidelity during replication, enabling rapid genetic changes and making them highly adaptable [6,7]. Secondly, CoVs utilize a distinct “copy–choice” mechanism in RNA replication, fostering the emergence of new strains and genotypes through homologous RNA recombination [8,9]. Finally, the substantial genome size of CoVs offers ample space for gene accommodation and modification, further augmenting their diversity and ability to adapt to new hosts and ecological niches [10]. This adaptability and plasticity of CoVs assume heightened significance when considering zoonotic spillover events, wherein these viruses can jump to new hosts and trigger extensive outbreaks with far–reaching consequences [11]. Therefore, gaining a comprehensive understanding of the breadth of diversity, genomics, and phylogeny of CoVs has become increasingly imperative in the context of emerging infectious diseases.

A specific focal point within CoV research revolves around the cophylogeny shared between bats and CoVs. Bats have been unequivocally identified as reservoirs for diverse CoVs [12], and their unique ecological and physiological attributes make them exceptional hosts for CoVs. Investigating the co-evolutionary interplay between bats and CoVs provides deeper insights into transmission dynamics, host adaptation, and evolutionary mechanisms underlying the emergence and dissemination of these viruses. Understanding these patterns is crucial for public health and the prevention of future pandemics, as it helps quantify spillover events and informs predictive models for zoonotic diseases.

Previous research has already demonstrated the illuminative potential of co-evolutionary investigations, uncovering invaluable insights into various occurrences such as host switching, cospeciation, and other events related to CoVs and their bat hosts [13]. However, despite these advancements, a comparative cophylogenetic analysis that delves into the discrete contributions of sequence fragments during host–pathogen co-evolution is noticeably lacking. To bridge this knowledge gap, the present study addresses this topic by examining the co-evolution of host–pathogen dynamics within bats and CoVs. Special attention is given to exploring potential correlations between the diversity of specific gene fragments of CoVs and the corresponding host topologies. This investigation is facilitated through the implementation of a sliding window strategy.

Our study encompassed geographically diverse locations across China, a nation renowned for its rich bat diversity, encompassing no fewer than 147 documented bat species [14]. This remarkable diversity firmly establishes China as one of the biodiversity hotspots of the world for bats, providing a crucial backdrop for our study. Notably, the prevalence of bat–borne viruses has sparked significant scientific interest in recent years, resulting in numerous dedicated studies aimed at comprehending the dynamics and consequences of CoVs within China [12,15,16]. By leveraging this extensive body of research and the wealth of sequencing data available, our investigation is well supported, emphasizing the significance of our exploration into the cophylogeny shared between bats and CoVs within this distinctive ecological context.

## 2. Materials and Methods

The sequence data used for analyses were obtained by conducting targeted searches across the online platforms PubMed and Google Scholar, employing keywords such as “bat” and “Coronavirus” in combination with “China”. Information regarding the virus–host association and geographical origins was referenced in the GenBank metadata or the original publications. Our dataset comprised 69 coronavirus (CoV) sequences isolated from various bat species in China, each identified at the species level. The bat species include *Aselliscus stoliczkanus*, *Chaerephon plicatus*, *Hipposideros pratti*, *Miniopterus fuliginosus*, *Miniopterus magnate*, *Miniopterus pusillus*, *Myotis ricketti*, *Pipistrellus abramus*, *Rhinolophus affinis*, *Rhinolophus blasii*, *Rhinolophus ferrumequinum*, *Rhinolophus macrotis*, *Rhinolophus pearsoni*, *Rhinolophus pusillus*, *Rhinolophus sinicus*, *Tylonycteris pachypus*, and *Vespertilio superans*. For molecular characterization, cytochrome b (cytb) gene sequences sourced from GenBank were utilized, as this mitochondrial gene is effective in achieving species–level resolution in mammalian phylogenies [17,18,19]. Altogether, these gene sequences were sourced from GenBank and encompass the complete genome, polyprotein 1ab (ORF1ab), spike sequences of the CoVs, and the cytb gene sequences from the bat specimens. Further information regarding these sequences is available in Table S1 in the Supplementary Materials and Github (https://github.com/tahiri-lab/aPhyloGeo.sm (accessed on 10 July 2024)).

### 2.1. Phylogenetic Analysis

Sequences of both CoVs and their respective host species were meticulously retrieved from GenBank. Subsequently, a comprehensive alignment of complete genomic sequences was conducted using the precision–oriented L–INS–I method within MAFFT (version 7.520) [20,21]. MAFFT is renowned for its accuracy and efficiency in handling large genomic sequences, making it ideal for nucleotide alignments. In contrast, MUSCLE is preferred for aligning protein–coding genes such as cytochrome b due to its capability to capture conserved protein motifs and structural features. The cytb genes of their hosts were aligned using the MUSCLE algorithm [22] with default parameters of the MegaX package [23]. A comprehensive evaluation of alignment accuracy was conducted, meticulously examining each alignment at a base–by–base level. The GBlocks (version 0.91.1) [24] from the NGPhylogeny.fr (https://ngphylogeny.fr/ (accessed on 10 July 2024)) web server [25,26] was used to eliminate sites with large proportions of gaps. Default parameters were used, except the “allowed gap positions” parameter, which was set to “With half”. Following these adjustments, multiple sequence alignments resulted in 1112 base pairs (bp) for cytb bat sequences, and 21,658 bp for CoVs’ complete genome sequences.

Maximum likelihood (ML) gene and genome phylogenies were inferred using the RAxML algorithm (version v0.9.0) [27]. Phylogenetic trees for both cytb and CoVs’ complete genome were constructed under the GTR + G + I DNA model in RAxML, based on their respective multiple sequence alignments. In each case, statistical support for branching patterns was estimated by bootstrap replication (100 replicates).

### 2.2. Cophylogeny Analysis between Host and Coronaviruses

To visualize the associations between hosts and CoVs, tanglegrams were generated based on the optimal ML trees. Global–fit methods were employed to quantify the congruence level between host and virus topologies, subsequently identifying specific associations that contribute to the cophylogenetic structure [28].

**Figure 1:**
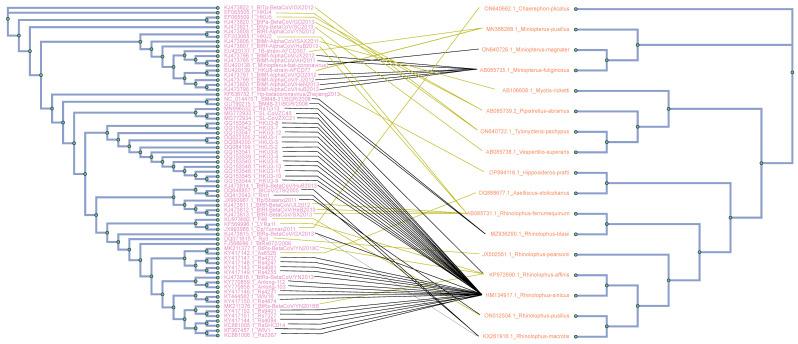
Tanglegram of cophylogenetic relationships between bat host and coronaviruses. Maximum likelihood phylogenies for coronaviruses (**left side**) and their bat hosts (**right side**), with bootstrap support values ≥75 labeled. All host–pathogen associations are shown in the tanglegram as dark yellow and black connecting lines. Black lines indicate significant individual cospeciation links between coronaviruses and their hosts, as indicated by both ParaFit and PACo (*p*-value ≤ 0.05), while dark yellow lines represent non–significant links.

The assessment of the global–fit analysis was conducted utilizing the distance–based ParaFit approach [29]. Patristic distance matrices were computed from maximum likelihood host and parasite phylogenies in R 3.0.1 [30]. Furthermore, ParaFit analyses were conducted in R using the “ape” package [31], and included 999 permutations to enable a global test, encompassing individual host–virus links. The significance of each host–virus interaction was determined through *p*-values of ParaFit1 or Parafit2 ≤ 0.05, represented by solid lines in the tanglegrams (see Figure 1). In acknowledgment of the inherent liberality of ParaFit, we also incorporated a methodology known as Procrustean Approach to Cophylogeny (PACo) [32] within the R environment. This approach involved the utilization of both the “ape” [31] and “vegan” [33] packages, enabling the acquisition of globally consistent goodness–of–fit statistics to validate the Parafit global values. PACo distinguishes itself from ParaFit by employing Procrustean superimposition, a technique that optimally adjusts the parasite matrix to align with the host matrix. Consequently, PACo directly assesses the degree of dependence of virus phylogeny on host phylogeny [34].

### 2.3. Comparison of Host and Coronavirus Phylogenies with Sliding Windows

Within the realm of significant host–virus interactions revealed by host and CoV phylogenies, we explored the potential correlations between specific gene fragment diversities of the virus and their corresponding host topologies. To undertake this analysis, we employed a sliding window approach [35,36] focusing on two pivotal CoV proteins: ORF1ab and spike.

**Figure 2:**
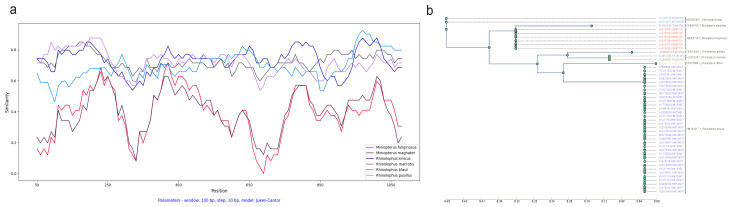
Cytochrome b (cytb) gene similarity and phylogenetic tree between *Rhinolophus affinis* and six other bat species: (**a**) SimPlot sliding window analysis of cytochrome b (cytb) gene similarity between *Rhinolophus affinis* and six other bat species; (**b**) cytochrome b (cytb) gene phylogenetic tree of seven bat species. Species clusters are indicated on the right. The seven–leaf tree was inferred using the RAxML method. To enable direct comparison with the coronavirus (CoV) tree, the branches of the original tree were meticulously duplicated for each bat species leaf, mirroring the count of collected CoV variants, and subsequently relabeled utilizing CoV variant descriptors. These labels integrate virus and host particulars with a hyphen, encompassing the NCBI genome accession number of the virus and its cytochrome b (cytb) gene accession number of host (Virus ID–Host ID).

Initially, we partitioned the multiple sequence alignment (MSA) of both ORF1ab and spike sequences into windows by stipulating a sliding window size (100 residues) and a step size (10 residues). Subsequently, we constructed a phylogenetic tree for each window. Concurrently, we performed a phylogenetic analysis of the relevant bat host, thereby creating a host tree as the reference tree. Crucially, the reference tree (based on host) and these sliding window trees (based on sliding windows) shared an identical set of leaves. To accomplish this, branches of the original reference tree were meticulously duplicated for each bat species leaf, mirroring the count of collected CoV variants, and subsequently relabeled utilizing CoV variant descriptors. These labels integrate virus and host particulars with a hyphen, encompassing the NCBI genome accession number of the virus and the cytochrome b (cytb) gene accession number of its host (Virus ID–Host ID) (see Figure 2b).

The association between the phylogenetic tree and the reference tree was then quantified using the Robinson–Foulds (RF) distance calculation (see Equation (1)) [37]. The RF distance fluctuation, determined via sliding windows, enabled the identification of gene fragments in which patterns of variation within virus species coincided with those of their respective hosts. This strategic approach enabled the evaluation of the contribution of each sequence fragment to the co-evolutionary relationship between the host and the pathogen. Fragments exhibiting lower RF distances played a more substantial role in cophylogeny, serving as informative reference points for future investigations. To ensure a streamlined and reproducible analytical process, we harnessed the computational workflow management system known as Snakemake [38] for this pipeline. This comprehensive pipeline consisted of three distinct stages: (1) alignment and sliding windows, (2) construction of phylogenetic trees for each window (virus tree) and for the host (reference tree), and (3) calculation of RF distance between virus tree and reference tree.

During the alignment and sliding window step, the multiple sequence alignment of ORF1ab and spike sequences was performed using the Clustal Omega (clustalo) algorithm [39]. Clustal Omega is widely used for aligning amino acid sequences due to its advanced algorithms optimized for protein alignment. The sliding window approach then extracted phylogenetic features from the alignment, systematically scanning the alignment with a window size of 100 residues moving from the 5′ (left) to the 3′ (right), with a fixed step size of 10 residues. The presence of mutations within each window was rigorously assessed, determining which windows proceeded to the subsequent analysis step based on the presence of at least one locus mutation.

For the construction of phylogenetic trees, we employed the RAxML–NG algorithm [40], an improved version of the well–known RAxML algorithm [27]. This facilitated the creation of phylogenetic trees for both sliding windows and the host cytb gene.

In the final step, the RF distance calculation quantified the dissimilarity between each pair of virus trees and the corresponding reference tree. Specifically, the RF distance between a virus tree ($T_{1}$) and reference tree ($T_{2}$) is the number of non–trivial bipartitions of $T_{1}$ that are not in $T_{2}$ plus the number of non–trivial bipartitions of $T_{2}$ that are not in $T_{1}$. This distance RF between $T_{1}$ and $T_{2}$, defined on the same set of leaves *n*, is computed by the following formula:

(1) $$RF\left(T_{1},T_{2}\right)=\frac{\left|(Q\backslash P)\cup (P\backslash Q)\right|}{2n-6},$$

where *Q* is a set containing all bipartitions in the virus tree (denoted $T_{1}$), *P* is a set containing all bipartitions in the reference tree (denoted $T_{2}$), and *n* the number of common leaves in $T_{1}$ or $T_{2}$. It is often relevant to normalize this distance by the maximum value of RF, which is equal to 2n−6 for two binary trees with *n* common leaves.

Structured upon the Snakemake workflow management system, our pipeline [41] efficiently executed diverse tasks using a range of tools and software. For instance, sequence downloading and data parsing were facilitated by the Entrez and SeqIO modules within Biopython v1.8.0 [42]. The raxml-ng v0.9.0 [40] enabled phylogenetic inference, while the Python 3.0 library robinson-foulds v1.1 based on the ETE toolkit [43] was employed for RF distance calculations. Additional Python libraries, such as NumPy [44] and pandas [45], were utilized for mutation testing. The comprehensive workflow can be accessed on Github (https://github.com/tahiri-lab/aPhyloGeo.sm (accessed on 10 July 2024)).

### 2.4. Similarity Analysis

The Python application SimPlot++ [46] served as a versatile tool for a range of analyses. These included identifying sequence similarity patterns, detecting both intergenic and intragenic recombination events using the Φ statistic [47], and generating as well as analyzing interactive sequence similarity networks.

Employing SimPlot++, we conducted a sliding window analysis to unveil patterns of sequence similarity. Our input data comprised multiple sequence alignments of ORF1ab and spike amino acid sequences (the same alignment files used in Section 2.3). These sequences underwent filtration through cophylogeny analysis and were subsequently grouped based on their host associations. Additionally, the multiple sequence alignment of the cytb gene was leveraged for analyzing the similarity of bat hosts. Among these similarity analyses, amino acid alignments were evaluated using the Kimura model, while the Jukes–Cantor model was applied to nucleotide sequence analysis. The Jukes–Cantor model assumes uniform substitution rates and a base composition for nucleotide sequences, suitable for closely related sequences and straightforward genetic distance calculations. In contrast, the Kimura model adjusts substitution rates based on amino acid chemical properties, providing a nuanced approach that considers protein structure and evolutionary dynamics, offering a realistic view of protein evolution [48].

### 2.5. Φ–Test Recombination Analysis

The Φ–test recombination analysis [49] was conducted to identify recombination patterns within the ORF1ab and spike genes of 47 CoVs. The Φ–profile program, integrated within the SimPlot++ application [46], was employed for this purpose. The input dataset consisted of the alignment of ORF1ab and spike amino acid sequences, which were the same alignment files used in the Section 2.3.

The Φ–profile program [46,47] identifies regions demonstrating the most distinct indications of mosaicism. It calculates the Φ statistic [49] utilizing a sliding window approach. The program provides both the Φ statistic value from a direct permutation test and the normal alternative to the permutation test [47]. Recognizing the influence of window size on the output, we executed the Φ–test recombination analysis for ORF1ab. The sliding windows ranged from 50 to 450 (in intervals of 50), with a breakpoint window set at 1000. Similarly, for the spike gene, the Φ–recombination test was conducted with sliding windows spanning from 50 to 200 (in intervals of 50) and a breakpoint window of 500. These configurations ensured an exhaustive analysis. For both tests, the window progress step was set to 10, and permutations were set to 1000.

### 2.6. Horizontal Gene Transfer Detection Analysis

We employed the horizontal gene transfer (HGT)–Detection program available on the T–Rex web server [50] to deduce directional horizontal gene transfer–recombination networks for the notable recombination regions spanning the ORF1ab and spike protein across 47 CoVs.

Trees generated from amino acid fragments, for which all the Φ–recombination tests yielded significant outcomes (≤0.05), were used as the gene trees. The rooted genome tree encompassing the 47 CoV viruses served as the foundational species tree. Each tree constructed from significant recombination regions was employed as input parameters for the HGT–Detection program. This tool facilitated the analysis of intergenic recombination events [51]. Certain transfers could potentially be attributed to the paradigm of parallel evolution, wherein species sharing akin environments experience similar mutations and develop corresponding traits.

The alignment and tree construction processes mirrored those of the phylogenetic analysis. The complete genome alignment was executed using MAFFT [20,21], while the alignment of amino acids was accomplished through Clustal Omega [39]. For tree construction, the RAxML-NG algorithm [40] was employed, supplemented by 100 bootstrap replicates.

## 3. Results

### 3.1. Phylogenetic Analysis

Phylogenetic analysis was conducted using a dataset comprising 69 complete genome sequences of bat CoVs, in addition to cytb gene sequences sourced from their respective hosts (see Figure 1). The phylogenetic trees for both viruses and bats were constructed using the RAxML algorithm, with bootstrap values computed from an ensemble of 100 trees. These trees were juxtaposed to illustrate their co-evolutionary patterns. Connections between hosts and pathogens are represented as lines linking the two sides of the tanglegram. Significant cospeciation events, where the evolutionary histories of bats and their associated coronaviruses are closely intertwined, are denoted by solid black lines. Conversely, non–significant associations between hosts and viruses are depicted by dark yellow lines. These connections indicate instances where the phylogenies of hosts and viruses do not clearly show simultaneous evolutionary divergence or co-evolutionary patterns. The outcomes obtained demonstrate strong and reliable phylogenetic support. Branches with bootstrap values ≥ 75 are accurately indicated on the provided tanglegram (see Figure 1).

### 3.2. Cophylogenetic Analysis between Hosts and CoVs

Global–fit methods were employed to assess the congruence among phylogenetic trees encompassing 17 bat species in China and the 69 variants of CoVs collected from them.

ParaFit analyses conducted on CoVs and bats have yielded compelling evidence that strongly supports the notion of substantial co-evolution between CoVs and their bat hosts. The calculated ParaFitGlobal value was determined to be 1934.133, with a statistically significant *p*-value of ≤0.001, underscoring the strength and validity of this observed co-evolutionary pattern.

Among the 69 individual host–parasite links, 64 exhibited significance with *p*-values of ParaFit1 and ParaFit2 ≤ 0.05. A notable majority of specific host–parasite connections (56) demonstrated strong significance, indicated by a low *p*-value of 0.001. This observation underscores the interdependence of phylogenetic relationships between bat species and CoV viruses. Consequently, it is plausible that the evolutionary history of bats has been influenced, at least partially, by interactions with CoVs, and vice versa. However, it is important to acknowledge that not all individual host–virus links exhibit statistically significant congruence, and there might be exceptions where co-evolution lacks robust support.

Similar outcomes were obtained through the Procrustean Approach to Cophylogeny (PACo) analysis. Jacknifed residuals resulting from applying PACo ranged from 0.02090178 to 0.22402673. For a more comprehensive analysis, we selected 47 CoVs–bat links with *p*-values lower than 0.05 in both ParaFit and PACo analyses. These 47 viruses were sourced from seven distinct bat species: (1) *Miniopterus fuliginosus* (six viruses), (2) *Miniopterus magnater* (one virus), (3) *Rhinolophus affinis* (one virus), (4) *Rhinolophus sinicus* (thirty–four viruses), (5) *Rhinolophus macrotis* (two viruses), (6) *Rhinolophus blasii* (two viruses), and (7) *Rhinolophus pusillus* (one virus).

### 3.3. Comparison of Host and CoV Phylogenies with Sliding Windows

Given the substantiated cophylogenetic relationship between bats and CoVs, an examination of crucial proteins, such as the amino acid sequences of ORF1ab and spike, can offer invaluable insights into the molecular interactions between the host and the virus. To identify specific regions or domains potentially involved in host–virus cophylogeny, we employed a sliding window approach to analyze the amino acid sequences of these two pivotal proteins (ORF1ab and spike) and evaluate their correlation with host topology.

Initially, we conducted a comparison of cytochrome b (cytb) gene similarity among the seven bat hosts using *Rhinolophus affinis* as a reference. Among these hosts, *Miniopterus fuliginosus* and *Miniopterus magnate* exhibited the highest similarity at 94%, while sharing only 78–80% similarity with other species. The average similarity among the *Rhinolophus* genus is 89.10%, ranging from 87% to 92% (see Figure 2a).

To explore the relationship between amino acid variations in ORF1ab and the corresponding host topology, we employed the calculation of the Robinson–Foulds (RF) distance between the phylogenetic tree of each sliding window and the phylogenetic tree representing the host topology (Table 1 and Table 2). This analysis mandates that the two computed trees share identical labels for the objects under comparison. Therefore, meticulous efforts were made to ensure label consistency between the phylogenetic tree of each sliding window and the phylogenetic tree representing the host topology. To achieve this alignment, branches of the original host tree were duplicated with precision for each bat species leaf. This duplication mirrored the count of collected CoV variants, and, subsequently, the duplicated branches were relabeled using CoV variant descriptors. These labels amalgamated specific virus and host details using a hyphen, encompassing the NCBI complete genome accession number of the virus and its cytochrome b (cytb) gene accession number host (Virus ID–Host ID), as depicted in Figure 2b.

**Figure 3:**
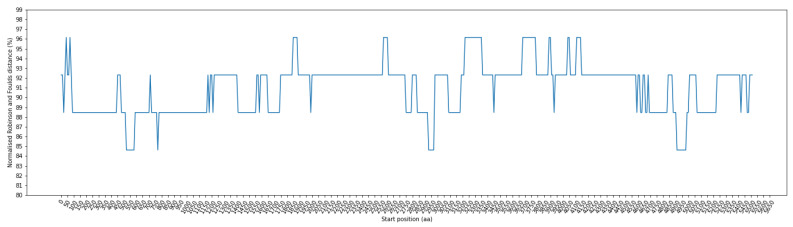
Fluctuation of normalized Robinson–Foulds (RF) distance across multiple sequence alignment (MSA) in phylogenetic trees: comparing coronavirus ORF1ab amino acids and bat cytochrome b (cytb) gene. The X–axis indicates the start position of sliding windows on the MSA.

Figure 3 illustrates the RF distance for each window position, employing a window size of 100 residues and a step size of 10 residues. Notably, these distances range from 84.62% to 96.15%. Of particular interest are the window ranges at positions 520–680, 770–870, 2930–3070, and 4910–5080, where the RF distance demonstrated its lowest value at 84.62%. To ensure the precise alignment of amino acid positions before and after the alignment step, we systematically removed indels. The positional information within the sequence before alignment can be found in Supplementary Table S2 for further details.

**Figure 4:**
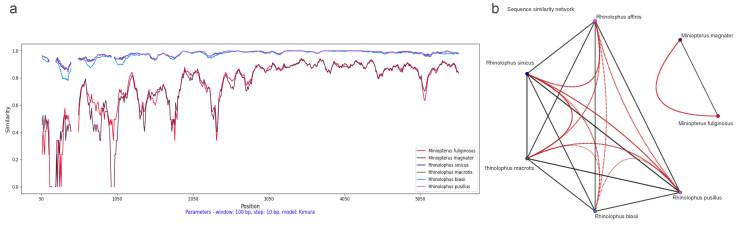
Dynamic sequence similarity patterns of ORF1ab amino acid sequences: (**a**) SimPlot sliding window analysis between coronaviruses from *Rhinolophus affinis* and six other bat species; and (**b**) sequence similarity network with global similarity threshold of 75%.

In addition, we performed a SimPlot analysis using amino acid sequences from the ORF1ab regions of 47 CoVs (see Figure 4). Guided by host associations, we categorized the 47 CoV variants into seven distinct groups (see Figure 2b). In the similarity analysis of the ORF1ab region, the *Miniopterus fuliginosus* and *Miniopterus magnate* groups displayed close proximity with 78% similarity, while sharing only 42–43% similarity with other groups (see Figure 4b). Using *Rhinolophus affinis* as reference, the *Rhinolophus pusillus* group demonstrated the highest similarity with the *Rhinolophus sinicus* group (98%), followed by similarity between the *Rhinolophus pusillus* and *Rhinolophus macrotis* groups (87%) (see Figure 4a).

**Figure 5:**
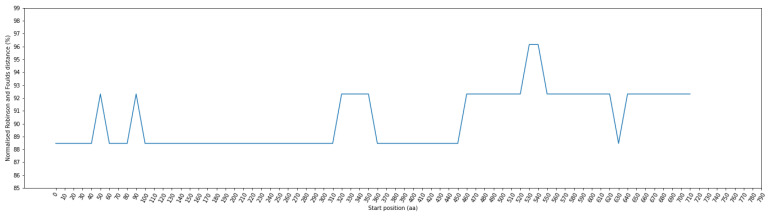
Fluctuation of normalized Robinson–Foulds (RF) distance across multiple sequence alignment (MSA) in phylogenetic trees: Comparing coronavirus spike amino acids and bat cytochrome b (cytb) gene. The X–axis indicates the start position of sliding windows on the MSA.

To explore the relationship between amino acid variations in the spike protein and their corresponding host topology, we calculated the RF distance between the phylogenetic tree of each sliding window and the phylogenetic tree representing the host topology. Figure 5 provides an illustration of RF distances for each window position, employing a window size of 100 residues and a step size of 10 residues. These RF distances range from 88.46% to 96.15%. Notably, among these positions, the window ranges at positions 0–140, 60–180, 100–410, 360–550, and 630–730 exhibit the lowest RF distance at 88.46%. The positional information within the sequence before alignment can be found in Supplementary Table S3 for further details.

**Figure 6:**
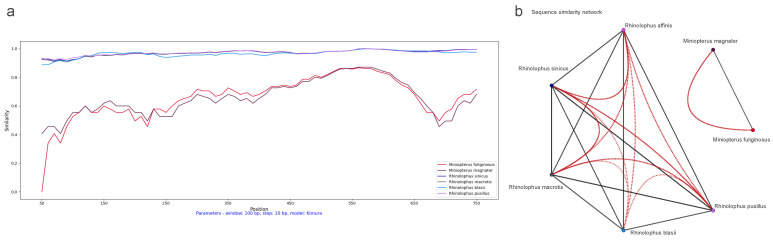
Dynamic sequence similarity patterns of spike amino acid sequences: (**a**) SimPlot sliding window analysis between coronaviruses from *Rhinolophus affinis* and six other bat species; and (**b**) sequence similarity network with global similarity threshold of 75%.

We also conducted a SimPlot analysis using amino acid sequences from the spike regions of 47 CoVs. Similar to the ORF1ab similarity analysis, we categorized the 47 CoV variants into seven distinct groups guided by their host associations (see Figure 2b). The similarity in the spike region was similar to the ORF1ab region, but with minor differences (see Figure 6). The *Miniopterus fuliginosus* and *Miniopterus magnate* groups exhibited close proximity with a 76% similarity, sharing only 29–30% similarity with other groups (see Figure 6b). Using *Rhinolophus affinis* as reference, the *Rhinolophus sinicus* and *Rhinolophus macrotis* groups shared the highest identity (97%), followed by the *Rhinolophus pusillus* group with the *Rhinolophus macrotis* group (95%) or the *Rhinolophus sinicus* group (95%) (see Figure 6a).

### 3.4. Detecting the Presence of Recombination

We employed a Φ–test recombination analysis [49] to investigate potential recent recombination events among amino acid sequences of CoVs. The Φ–test was conducted with various sliding window sizes. Specifically, for longer genes like the ORF1ab sequence, the window sizes ranged from 50 to 450 with an interval of 50, encompassing sizes such as 50, 100, 150, 200, 250, 300, 350, 400, and 450. Conversely, for shorter genes like the spike sequence, the window sizes ranged from 50 to 200, covering sizes of 50, 100, 150, and 200. The window advancement interval was set at 10. The selection of the window size was meticulously considered as it can influence the test outcomes.

The results, presented in Table 3, correspond to the window positions of the ORF1ab amino acid sequence with *p*-values ≤ 0.05 in all window size configurations. A threshold of *p*-values ≤ 0.05 was deemed significant, indicating potential recombination within the examined window position. Among the tested window positions, 31 within ORF1ab suggested the presence of recombination events. Merging adjacent windows revealed six significant recombination regions, corresponding to alignment positions 360–1390, 550–1610, 680–1680, 700–1710, 2060–3090, and 2130–3250.

The same Φ–test recombination analysis was performed for the amino acid sequence of the spike protein (see Table 4). A total of nine window positions consistently displayed lower *p*-values (≤0.05). By merging adjacent windows, we identified four significant recombination regions, corresponding to alignment positions 10–510, 50–560, 170–710, and 230–730.

As the calculations for RF, similarity, and Φ utilized identical input alignments, the regions pinpointed in these outcomes are directly comparable. Among the regions highlighted with the lowest RF distance, except for the position 4910–5080 in ORF1ab, all other regions aligned with noteworthy recombination regions (see Table 1 and Table 2).

### 3.5. Horizontal Gene Transfer Analyses

In our study, we employed a comprehensive range of analytical approaches to investigate the evolutionary relationships and instances of horizontal gene transfer (HGT) among 47 CoV variants. In addition to employing SimPlot similarity and Φ–test recombination analyses, we conducted a more comprehensive analysis to detect potential HGT events among the notable recombination regions within the ORF1ab and spike segments.

**Figure 7:**
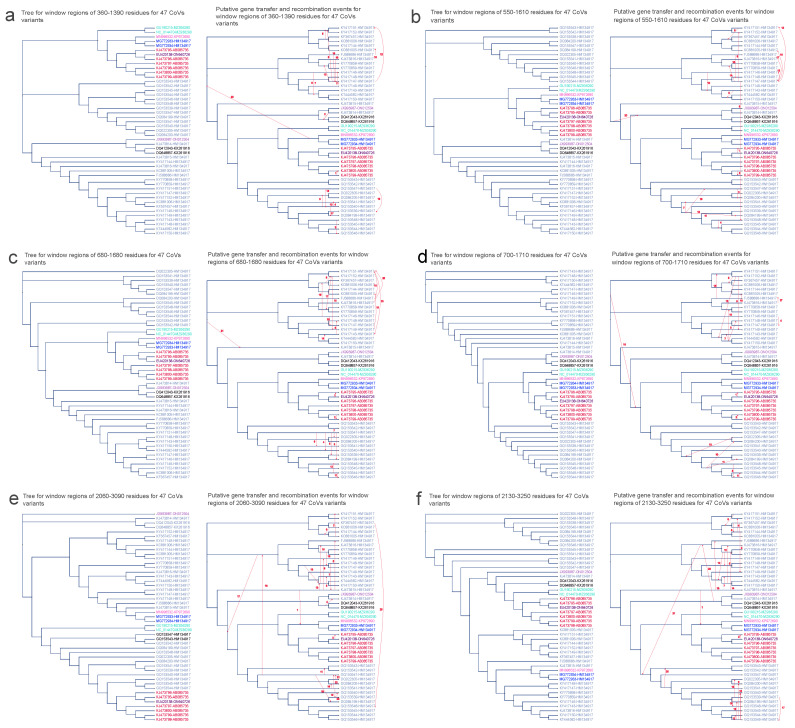
Putative horizontal gene transfer events found for the window regions of ORF1ab amino acid sequences of 47 CoV variants. The left segment of each figure section displays the tree generated from significant recombination regions, while the corresponding right section depicts the species tree (referred to as the whole genome tree) wherein statistically significant horizontal gene transfers (HGT) are graphically represented using arrows to indicate transfer directions. The figure shows 6 significant recombination regions: panel (**a**) between 360 and 1390 residues, panel (**b**) between 550 and 1610 residues, panel (**c**) between 360 and 1680 residues, panel (**d**) between 700 and 1710 residues, panel (**e**) between 2060 and 3090 residues, and panel (**f**) between 2130 and 3250 residues. CoV variant labels integrate virus and host particulars with a hyphen, encompassing the NCBI genome accession number of the virus and its cytochrome b (cytb) gene accession number of the host (Virus ID–Host ID). Distinct hosts are distinguished by various colors: AB085735 signifies *Miniopterus fuliginosus* in red; ON640726 represents *Miniopterus magnater* in maroon; KP972690 corresponds to *Rhinolophus affinis* in magenta; HM134917 denotes *Rhinolophus sinicus* in blue; KX261916 signifies *Rhinolophus macrotis* in gray; MZ936290 corresponds to *Rhinolophus blasii* in aqua; and ON012504 stands for *Rhinolophus pusillus* in mauve.

**Figure 8:**
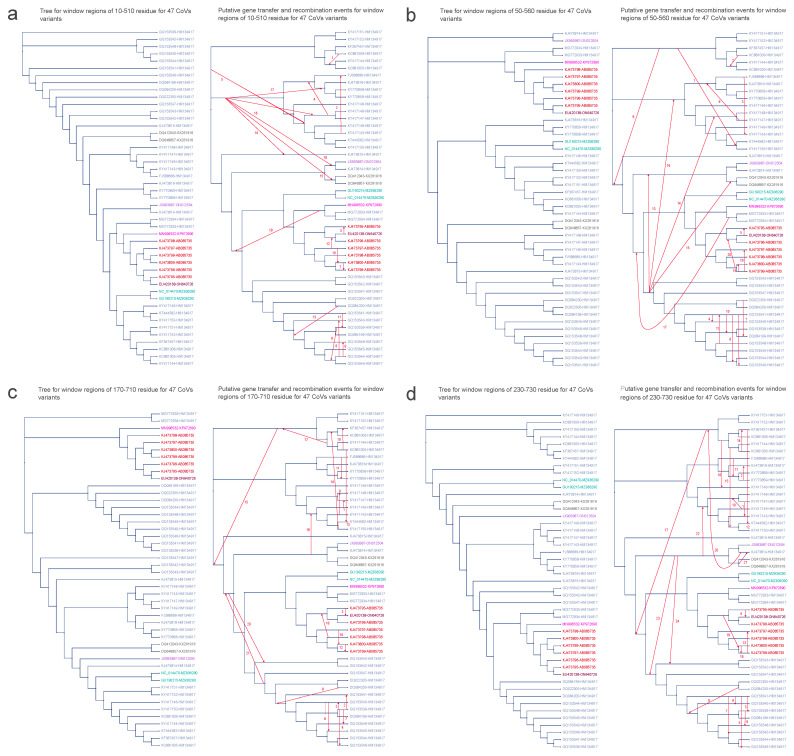
Putative horizontal gene transfer events found for the window regions of spike amino acid sequences of 47 CoV variants. The left segment of each figure section displays the tree generated from significant recombination regions, while the corresponding right section depicts the species tree (referred to as the whole genome tree) wherein statistically significant horizontal gene transfers are graphically represented using arrows to indicate transfer directions. The figure shows 4 significant recombination regions: panel (**a**) between 10 and 510 residues, panel (**b**) between 50 and 560 residues, panel (**c**) between 170 and 710 residues, and panel (**d**) between 230 and 730 residues. CoV variant labels integrate virus and host particulars with a hyphen, encompassing the NCBI genome accession number of virus and its cytochrome b (cytb) gene accession number of host (Virus ID–Host ID). Distinct hosts are distinguished by various colors: AB085735 signifies *Miniopterus fuliginosus* in red; ON640726 represents *Miniopterus magnater* in maroon; KP972690 corresponds to *Rhinolophus affinis* in magenta; HM134917 denotes *Rhinolophus sinicus* in blue; KX261916 signifies *Rhinolophus macrotis* in gray; MZ936290 corresponds to *Rhinolophus blasii* in aqua; and ON012504 stands for *Rhinolophus pusillus* in mauve.

Firstly, we performed complete genome phylogeny inference for the 47 CoV variants, along with constructing individual amino acid fragment trees for noteworthy recombination regions within the ORF1ab and spike segments. For these phylogenetic analyses, we employed the RAxML–NG method and incorporated bootstrapping to ensure robust statistical support. Subsequently, an extensive HGT analysis (see Figure 7 and Figure 8) was conducted using the HGT–Detection program on the T–Rex web server [50]. This process involved reconciling the rooted genome tree that encompassed the 47 CoV organisms as the foundational species tree. We used each tree generated from significant recombination regions as input parameters for the HGT–Detection program. We discerned potential HGT events among CoV variants by comparing the species tree with various gene phylogenies for specific regions. Our focus was on the ten regions identified in the Φ–recombination test, which included six from ORF1ab and four from spike. Significant insights emerged from the interpretation of each identified HGT event, allowing for three possible scenarios: (1) a distinct complete or partial HGT event connecting distant species; (2) the result of parallel evolution among the implicated species; or (3) the emergence of a novel species (referred to as the recipient). This emergence arises due to the recombination between the genome of the donor species and that of a neighboring recipient species in the species phylogeny [52].

We now shift our attention to the evolution of six crucial amino acid fragments within the ORF1ab protein, a substantial segment of CoV genomes encompassing over two–thirds of their total length. The ORF1ab gene encodes the replicase polyprotein, synthesized through the translation of ORF1a and ORF1b [53]. This gene plays a pivotal role in virus pathogenesis [54]. Remarkably, most partial gene transfer events within this gene were detected among virus variants from the same host (see Figure 7). This observation is consistent with the fact that multiple variants can coexist within a single host, potentially allowing for the exchange of genetic information between these variants. Additionally, intriguingly, HGT events between variants of different hosts were also identified. For instance, HGT between the variant from *Miniopterus magnater* (EU420138–ON640726) and the variant from *Miniopterus fuliginosus* (KJ473795–AB085735) were postulated within regions 550–1610, 680–1680, 700–1710, 2060–3090, and 2130–3250 (see Figure 7b–f). Furthermore, HGT events were postulated between the variant from *Rhinolophus pusillus* (JX993987–ON012504) and variants from *Rhinolophus macrotis* (KX261916) within the region 2060–3090 (see Figure 7e), as well as between the variant from *Rhinolophus pusillus* (JX993987-ON012504) and variants from *Rhinolophus sinicus* (HM134917) within the region 2130–3250 (see Figure 7f).

The spike protein, commonly referred to as the S glycoprotein, encompasses both the receptor-binding domain (RBD) and fusion-related domains, establishing it as the central protein in the CoV entry process [55]. The trimeric configurations of the spike proteins on the virion surface promote attachment to cell surface receptors and facilitate the fusion of viral and cell membranes [56]. Consequently, the viral spike entry protein serves as the primary determinant of CoV tropism. Beyond its pivotal role in CoV host tropism and pathogenesis, the spike protein serves as the primary target for neutralizing antibodies generated by the immune system of the infected host [57]. In summary, the spike protein appears to be the key factor influencing the success of initial cross–species infection events. In this study, we conducted HGT analyses on four essential amino acid fragments within the spike protein.

Similar to the observations in ORF1ab, the majority of instances of partial HGT events within the spike protein were identified among virus variants originating from the same host (see Figure 8). Consistent with the findings of ORF1ab, HGT between the variant from *Miniopterus magnater* (EU420138–ON640726) and the variant from *Miniopterus fuliginosus* (KJ473795–AB085735) were detected across all four regions of spike: (1) 10–510, (2) 50–560, (3) 170–710, and (4) 230–730 (see Figure 8a–d). Regarding HGT involving variants from *Rhinolophus macrotis* (KX261916), the identified recombination partner in spike differed from that in the ORF1ab results. Within the region 230–730 (see Figure 8d), instead of JX993987–ON012504 (a variant from *Rhinolophus pusillus*), it was KJ473814–HM134917 (a variant from *Rhinolophus sinicus*) that exhibited recombination with KX261916. This outcome might arise from the similarity between KJ473814–HM134917 and JX993987–ON012504, a similarity underscored by their shared placement within the same branch on the phylogenetic tree. Similarly, within the region 170–710 (see Figure 8c), a substitution occurred in the HGT involving variants from *Rhinolophus sinicus* (HM134917), where KJ473814–HM134917 replaced JX993987–ON012504 as the recombination partner (see Supplementary Tables S2 and S3 for further details).

## 4. Discussion

When contemplating the associations between hosts and parasites, particularly within the scope of our study of CoVs, an analogy can be drawn from vicariance biogeography. In this analogy, the host can be likened to a “region” exploited by the parasite, and host speciation can be seen as analogous to a “vicariance event” [58]. To ascertain whether closely related hosts exhibit a stronger correlation with similar CoV variants than anticipated by chance, we employed global–fit methods. Our analysis focused on 17 bat species in China and the collection of 69 CoV variants derived from them. Notably, our analysis identified a significant cophylogenetic signal involving seven bat species and their corresponding 49 CoV variants.

To explore the cophylogenetic dynamics further, we compared host and CoV phylogenies using the RF distance metric, and examined recombination events among variants using the Φ–test. By employing a sliding window approach, the RF distance analysis allowed us to delve into the contribution of each genomic segment to the cophylogenetic dynamics. The Φ–test pinpointed regions within key protein sequences that underwent significant genetic exchanges, impacting overall diversity. We also investigated horizontal gene transfer (HGT) associated with each recombination region, considering both host–virus similarity and diversity introduced by recombination.

The foundation of genetic diversity rests primarily on two fundamental processes: mutations and recombination. Mutations induce changes in nucleotide states, leading to the emergence of new variants. On the other hand, recombination facilitates the transfer of variants across genomes, ultimately giving rise to novel haplotypes [59]. Recombination is pervasive among the majority of viruses, wherein independently arising variants coalesce within the same molecular structure, providing viruses with fresh avenues to navigate selective pressures and successfully adapt to novel environments and hosts [60]. Our analysis has illuminated the frequent occurrence of recombination among CoVs originating from the same host species.

Genome sequencing and phylogenetic investigations reveal that, while individual virus species typically exhibit a narrow host range, often limited to a single animal species, there is frequent interspecies transmission of CoVs [61,62]. In this context, recombination and re-assortment function as potent mechanisms allowing viruses to acquire novel antigenic combinations, thereby facilitating their adaptation to new host environments. However, ascribing the emergence of a specific RNA virus solely to its recombination ability is often a complex undertaking, with notable exceptions like the Western equine encephalitis virus [63] and the turkey coronaviruses [64] serving as direct examples where recombination plays a pivotal role in emergence.

Recombination, a prevalent phenomenon across viruses, holds significant sway over their evolutionary trajectory. Its impact extends to widening viral host ranges, generating novel virus strains, enhancing virulence and pathogenesis, modifying tissue tropisms, evading host immunity, and fostering resistance to antiviral strategies [65,66]. Despite its relatively infrequent occurrence, our study illuminates instances of recombination within CoVs originating from various bat species within the same genus. Interestingly, our investigation uncovers not only intraspecies recombination within variants from the same host species but also interspecies recombination spanning different host species. For instance, within the ORF1ab region, we detected transfers between variants from *Miniopterus magnater* (EU420138-ON640726) and *Miniopterus fuliginosus* (KJ473795-AB085735) across regions 550–1610, 680–1680, 700–1710, 2060–3090, and 2130–3250. Furthermore, transfers between the aforementioned variants were identified across all four spike protein regions: (1) 10–510, (2) 50–560, (3) 170–710, and (4) 230–730. These instances of horizontal gene transfer between hosts merit deeper exploration through meticulous biological experimentation.

Our comprehensive exploration of the cophylogenetic relationship between bat hosts and CoVs has shed light on the intricate molecular interactions that govern this dynamic association. Through rigorous phylogenetic analyses, we have revealed compelling evidence of substantial cophylogeny between CoVs and their bat hosts, substantiated by ParaFit analyses and PACo results. This intricate interplay between host and virus highlights the reciprocal impact of bat hosts on CoV diversification. The observed congruence emphasizes the importance of examining multiple layers of evidence to comprehensively understand cophylogeny.

The detailed examination of critical proteins, particularly the amino acid sequences of ORF1ab and spike, has provided valuable insights into the potential regions contributing to the cophylogenetic dynamics between hosts and viruses. By employing sliding window methodologies, we have identified specific window positions within the ORF1ab and spike sequences that indicate the occurrence of recombination events, highlighting the adaptive nature of these interactions.

Employing a sliding window approach and RF distance, we scrutinized the contribution of each genomic segment to the cophylogenetic process. On the other hand, the Φ-test for recombination focused on identifying regions within key protein sequences that underwent substantial genetic material exchanges, consequently influencing diversity. Four regions on ORF1ab, as well as five regions on spike, were detected as contributing more to the participant host–virus cophylogeny than other regions within the gene. Worthy of note, significant recombination was detected in eight of these nine regions. This suggests that CoVs may promote their adaptation to their hosts through recombination. Based on this investigation, we further inferred the horizontal gene transfer (HGT) process associated with each recombination region. HGT analysis uncovers not only intraspecies recombination within variants from the same host species but also interspecies recombination spanning different host species.

The study included the comparison and analysis of only 69 CoVs sequences from 17 bat species in China. By focusing on a specific region, the study offered targeted insights into the diversity, evolution, and host–pathogen interactions within the Chinese bat population, though it may not fully generalize to global bat diversity. This limited dataset highlights the need for further investigation into the evolution of CoVs in other bat species around the world to obtain a more comprehensive understanding of the dynamics and co-evolution between bats and CoVs on a broader scale. To enhance future studies, it would be beneficial to expand the dataset.

While our findings establish a robust framework for understanding the cophylogeny between bats and CoVs, the intricate nature of this relationship calls for further investigations to unveil underlying mechanisms. For instance, there is potential for deeper investigation into the structure and function of the active recombination fragments identified in this study. Moreover, new studies could explore the existence of inter–genus recombination of CoVs.

## 5. Conclusions

Our work contributes to a broader understanding of the intricate interactions that shape the cophylogenetic landscape between bat hosts and CoVs. By dissecting these relationships at the genetic and phylogenetic levels, we aim to cultivate a deeper appreciation of the underlying mechanisms driving viral diversity and host adaptation. Ultimately, this knowledge informs our response to current and future challenges posed by infectious diseases. In a world grappling with emerging viral diseases, a deeper understanding of the cophylogenetic dynamics between hosts and viruses could offer crucial insights for surveillance, prevention, and management strategies.

## Figures and Tables

**Table 1:** Percentage of normalized Robinson–Foulds (RF) distance between each pair of phylogenetic trees based on coronavirus ORF1ab amino acids and bat cytochrome b (cytb) gene.

Start Position of Window (aa)	End Position of Window (aa)	RF Distance (%)	Recombination Detected (Yes/No)
520	680	84.62	yes
770	870	84.62	yes
2930	3070	84.62	yes
4910	5080	84.62	no

**Table 2:** Percentage of normalized Robinson–Foulds (RF) distance between each pair of phylogenetic trees based on coronavirus spike amino acids and bat cytochrome b (cytb) gene.

Start Position of Window (aa)	End Position of Window (aa)	RF Distance (%)	Recombination Detected (Yes/No)
0	140	88.46	yes
60	180	88.46	yes
100	410	88.46	yes
360	550	88.46	yes
630	730	88.46	yes

**Table 3:** The results of the Φ recombination test carried out for the ORF1ab amino acid sequence of the 47 CoVs.

Number of Region	Start Position of Break Window (aa)	End Position of Break Window (aa)	Φ–Test Result (*p*-Value)	Window Size
1	360	1360	$4.18\times 10^{-3}$	150
	370	1370	$1.84\times 10^{-3}$	150
	380	1380	$3.96\times 10^{-4}$	150
	390	1390	$7.82\times 10^{-4}$	450
2	550	1550	$2.15\times 10^{-6}$	350
	560	1560	$9.20\times 10^{-7}$	300
	570	1570	$1.79\times 10^{-7}$	300
	580	1580	$5.20\times 10^{-7}$	300
	590	1590	$2.08\times 10^{-7}$	300
	600	1600	$1.05\times 10^{-6}$	300
	610	1610	$2.74\times 10^{-5}$	300
3	680	1680	$8.51\times 10^{-4}$	250
4	700	1700	$5.31\times 10^{-3}$	250
	710	1710	$7.87\times 10^{-3}$	250
5	2060	3060	$3.57\times 10^{-3}$	300
	2070	3070	$3.10\times 10^{-3}$	300
	2080	3080	$3.77\times 10^{-3}$	300
	2090	3090	$2.15\times 10^{-3}$	300
6	2130	3130	$3.30\times 10^{-3}$	400
	2140	3140	$1.46\times 10^{-3}$	200
	2150	3150	$8.79\times 10^{-4}$	200
	2160	3160	$5.48\times 10^{-4}$	200
	2170	3170	$1.66\times 10^{-4}$	150
	2180	3180	$2.38\times 10^{-5}$	100
	2190	3190	$1.81\times 10^{-5}$	100
	2200	3200	$6.19\times 10^{-7}$	100
	2210	3210	$4.57\times 10^{-5}$	100
	2220	3220	$1.07\times 10^{-10}$	100
	2230	3230	$2.49\times 10^{-7}$	100
	2240	3240	$8.10\times 10^{-7}$	100
	2250	3250	$1.01\times 10^{-6}$	100

**Table 4:** The results of the Φ recombination test carried out for the spike amino acid sequence of the 47 CoVs.

Number of Region	Start Position of Break Window (aa)	End Position of Break Window (aa)	Φ–Test Result (*p*-Value)	Window Size
1	10	510	$4.82\times 10^{-4}$	50
2	50	550	$4.97\times 10^{-4}$	50
	60	560	$4.46\times 10^{-4}$	50
3	170	670	$1.28\times 10^{-3}$	50
	180	680	$9.60\times 10^{-4}$	50
	190	690	$6.35\times 10^{-4}$	50
	200	700	$1.42\times 10^{-4}$	50
	210	710	$7.81\times 10^{-3}$	150
4	230	730	$7.19\times 10^{-3}$	50

## Data Availability

A preliminary open–source implementation of our algorithm and all datasets (simulated and biological datasets) are freely available on GitHub at https://github.com/tahiri-lab/aPhyloGeo.sm, accessed on 10 July 2024.

## Supplementary Materials

The following supporting information can be downloaded at https://www.mdpi.com/article/10.3390/v16071133/s1; Table S1: Coronaviruses and bats sequences used for cophylogenetic analyses; and Table S2: Positions of windows on alignment and HKU3–6 for Lower RF and HGT, Table S3: Positions of windows on alignment and HKU3–6 for Lower RF and HGT, and Table S4: Virus and Host Data.

## Abbreviations

The following abbreviations are used in this manuscript:

aa	Amino acid
bp	Base pair
CoVs	Coronaviruses
cytb	Cytochrome b
DNA	DeoxyriboNucleic acid
HGT	Horizontal gene transfer
NCBI	National Center for Biotechnology Information
MAFFT	Multiple Alignment using Fast Fourier Transform
ML	Maximum likelihood
MSA	Multiple sequence alignment
MUSCLE	MUltiple Sequence Comparison by Log–Expectation
ORF1ab	Open Reading Frames 1a and b
RAxML–NG	Randomized Axelerated Maximum Likelihood Next Generation
RF	Robinson–Foulds distance
RNA	RiboNucleic acid
