# Supplementary material for: Host–Virus Cophylogenetic Trajectories: Investigating Molecular Relationships between Coronaviruses and Bat Hosts
Source: Viruses. 2024 Jul 15;16(7):1133. doi: 10.3390/v16071133 (PMC11281392; doi:10.3390/v16071133)
Supplement: Supplementary file 1 [file viruses-16-01133-s001.zip › viruses-3066851-supplementary.pdf]

Host-Virus Cophylogeny Trajectories: Investigating Molecular Relationships between Coronaviruses and Bat Hosts

Wanlin Li and Nadia Tahiri

Department of Computer Science, University of Sherbrooke, 2500 Bd University, Sherbrooke, Quebec, Canada
Correspondence: Nadia.Tahiri@USherbrooke.ca

The dataset includes sequences from 69 distinct CoV genotypes, which have their origins in 17 different bat species. These genetic sequences have been obtained from GenBank and encompass the complete genome, polyprotein 1ab (ORF1ab), spike sequences of the CoVs, and the cytb gene sequences from the bat specimens.

The 17 bat hosts include individuals in the *Aselliscus stoliczkanus*, *Chaerephon plicatus*, *Hipposideros pratti*, *Miniopterus fuliginosus*, *Miniopterus magnate*, *Miniopterus pusillus*, *Myotis ricketti*, *Pipistrellus abramus*, *Rhinolophus affinis*, *Rhinolophus blasii*, *Rhinolophus ferrumequinum*, *Rhinolophus macrotis*, *Rhinolophus pearsoni*, *Rhinolophus pusillus*, *Rhinolophus sinicus*, *Tylonycteris pachypus*, and *Vespertilio superans*. For molecular characterization, cytochrome b (cytb) gene sequences from all sampled bats were sourced from GenBank. This mitochondrial gene has proven instrumental in achieving species-level resolution for mammalian phylogenies within the Order [1, 2, 3].

Table S1: Coronaviruses and bats sequences used for cophylogenetic analyses

**Virus complete genome**	**Virus ORF1ab**	**Virus spike**	**Host**	**Host cytb**	**Reference**
KY417142	ATO98106	ATO98108	Aselliscus stoliczkanus	DQ888677	[4, 5]
JX993988	AGC74171(1a); AGC74177(1b)	AGC74176	*Chaerephon plicatus*	ON640662	[6, 7]
KF636752	AIL94214	AIL94216	*Hipposideros pratti*	OP894116	[8]
KJ473795	AIA62199	AIA62200	*Miniopterus fuliginosus*	AB085735	[9, 10]
KJ473796	AIA62205	AIA62206	*Miniopterus fuliginosus*	AB085735	[9, 10]
KJ473797	AIA62211	AIA62212	*Miniopterus fuliginosus*	AB085735	[9, 10]
KJ473798	AIA62219	AIA62220	*Miniopterus fuliginosus*	AB085735	[9, 10]
KJ473799	AIA62226	AIA62227	*Miniopterus fuliginosus*	AB085735	[9, 10]
KJ473800	AIA62233	AIA62234	*Miniopterus fuliginosus*	AB085735	[9, 10]
EU420138	ACA52163	ACA52164	*Miniopterus magnater*	ON640726	[7, 11]
EU420137	ACA52156	ACA52157	*Miniopterus pusillus*	MN366288	[11]
EU420139	ACA52170	ACA52171	*Miniopterus pusillus*	MN366288	[11]
KJ473806	AIA62245	AIA62246	*Myotis ricketti*	AB106608	[12, 13]
KJ473820	AIA62342	AIA62343	*Pipistrellus abramus*	AB085739	[8, 10]
EF065509	ABN10874	ABN10875	*Pipistrellus abramus*	AB085739	[10, 14]
KF569996	AHX37556(1a); AHX37557(1b)	AHX37558	*Rhinolophus affinis*	KP972690	[14]
MK211376	QDF43824	QDF43825	*Rhinolophus affinis*	KP972690	[14, 15]
MK211377	QDF43829	QDF43830	*Rhinolophus affinis*	KP972690	[14, 15]
MN996532	QHR63299	QHR63300	*Rhinolophus affinis*	KP972690	[14, 16]
GU190215	ADK66840	ADK66841	*Rhinolophus blasii*	MZ936290	[17, 18]
NC014470	YP003858583	YP003858584	*Rhinolophus blasii*	MZ936290	[17, 18]
KJ473807	AIA62251	AIA62252	*Rhinolophus ferrumequinum*	AB085731	[10, 12]
KJ473808	AIA62258	AIA62259	*Rhinolophus ferrumequinum*	AB085731	[10, 12]
KJ473811	AIA62276	AIA62277	*Rhinolophus ferrumequinum*	AB085731	[10, 12]
KJ473812	AIA62289	AIA62290	*Rhinolophus ferrumequinum*	AB085731	[10, 12]
KJ473813	AIA62299	AIA62300	*Rhinolophus ferrumequinum*	AB085731	[10, 12]

Continued on next page

Table S1 – continued from previous page

Virus complete genome	Virus ORF1ab	Virus spike	Host	Host cytb	Reference
DQ412043	ABD75330(1a); ABD75331(1b)	ABD75332	*Rhinolophus macrotis*	KX261916	[19]
DQ648857	ABG47068	ABG47069	*Rhinolophus macrotis*	KX261916	[19]
DQ071615	AAZ67050(1a); AAZ67051(1b)	AAZ67052	*Rhinolophus pearsoni*	JX502551	[20]
JX993987	AGC74164(1a); AGC74170(1b)	AGC74165	*Rhinolophus pusillus*	ON012504	[6, 21]
KU973692	ARO76381(1a)	ARO76382	*Rhinolophus pusillus*	ON012504	[6, 21]
DQ022305	AAY88865	AAY88866	*Rhinolophus sinicus*	HM134917	[22]
DQ084199	AAZ41328	AAZ41329	*Rhinolophus sinicus*	HM134917	[22]
DQ084200	AAZ41339	AAZ41340	*Rhinolophus sinicus*	HM134917	[22]
FJ588686	ACU31044	ACU31032	*Rhinolophus sinicus*	HM134917	[22, 23]
GQ153539	ADE34721	ADE34722	*Rhinolophus sinicus*	HM134917	[22, 23]
GQ153540	ADE34732	ADE34733	*Rhinolophus sinicus*	HM134917	[22, 23]
GQ153541	ADE34743	ADE34744	*Rhinolophus sinicus*	HM134917	[22, 23]
GQ153542	ADE34754	ADE34755	*Rhinolophus sinicus*	HM134917	[22, 23]
GQ153543	ADE34765	ADE34766	*Rhinolophus sinicus*	HM134917	[22, 23]
GQ153544	ADE34778	ADE34779	*Rhinolophus sinicus*	HM134917	[22, 23]
GQ153545	ADE34789	ADE34790	*Rhinolophus sinicus*	HM134917	[22, 23]
GQ153546	ADE34800	ADE34801	*Rhinolophus sinicus*	HM134917	[22, 23]
GQ153547	ADE34811	ADE34812	*Rhinolophus sinicus*	HM134917	[22, 23]
GQ153548	ADE34822	ADE34823	*Rhinolophus sinicus*	HM134917	[22, 24]
KC881005	AGZ48805	AGZ48806	*Rhinolophus sinicus*	HM134917	[25, 22]
KC881006	KC881006	AGZ48818	*Rhinolophus sinicus*	HM134917	[25, 22]
KF367457	AGZ48830	AGZ48831	*Rhinolophus sinicus*	HM134917	[25, 22]
KJ473814	AIA62309	AIA62310	*Rhinolophus sinicus*	HM134917	[8, 22]
KJ473815	AIA62319	AIA62320	*Rhinolophus sinicus*	HM134917	[8, 22]
KJ473816	AIA62329	AIA62330	*Rhinolophus sinicus*	HM134917	[8, 22]
KT444582	ALK02468	ALK02457	*Rhinolophus sinicus*	HM134917	[26, 22]
KY417143	ATO98118	ATO98120	*Rhinolophus sinicus*	HM134917	[4, 22]
KY417144	ATO98130	ATO98132	*Rhinolophus sinicus*	HM134917	[4, 22]

Continued on next page

Table S1 – continued from previous page

Virus complete genome	Virus ORF1ab	Virus spike	Host	Host cytb	Reference
KY417146	ATO98155	ATO98157	*Rhinolophus sinicus*	HM134917	[4, 22]
KY417147	ATO98167	ATO98169	*Rhinolophus sinicus*	HM134917	[4, 22]
KY417148	ATO98179	ATO98181	*Rhinolophus sinicus*	HM134917	[4, 22]
KY417149	ATO98191	ATO98193	*Rhinolophus sinicus*	HM134917	[4, 22]
KY417150	ATO98203	ATO98205	*Rhinolophus sinicus*	HM134917	[4, 22]
KY417151	ATO98216	ATO98218	*Rhinolophus sinicus*	HM134917	[4, 22]
KY417152	ATO98229	ATO98231	*Rhinolophus sinicus*	HM134917	[4, 22]
KY770858	ARI44798	ARI44799	*Rhinolophus sinicus*	HM134917	[22]
KY770859	ARI44803	ARI44804	*Rhinolophus sinicus*	HM134917	[22]
MG772933	AVP78030	AVP78031	*Rhinolophus sinicus*	HM134917	[22]
MG772934	AVP78041	AVP78042	*Rhinolophus sinicus*	HM134917	[22]
EF203065	ABQ57215	ABQ57216	*Rhinolophus sinicus*	HM134917	[22]
KJ473822	AIA62351	AIA62352	*Tylonycteris pachypus*	ON640722	[12]
EF065505	ABN10838	ABN10839	*Tylonycteris pachypus*	ON640722	[14]
KJ473821	AHY61336	AHY61337	*Vespertilio superans*	AB085738	[8, 10]

The positions showing cophylogenetic association and recombination were calculated based on the window positions after multiple sequence alignments. Due to the presence of insertions and deletions during the multiple sequence alignment process, we selected the sequence of sample HKU3-6 as the reference sequence. We then reverse-mapped the results of the multiple sequence alignment to their actual positions in the HKU3-6 sample. The specific correspondence is shown in the tables below.

	**Position on alignment (aa)**	**Position on HKU3-6 (aa)**
**Lower RF**	520-680	894-1136
	770-870	1478-1635
	2930-3070	4279-4501
	4910-5080	6366-6544
**HGT**	360-1390	670-2585
	550-1610	924-2859
	680-1680	1136-2943
	700-1710	1163-2974
	2060-3090	3350-4521
	2130-3250	3420-4681

Table S2: Positions of windows on alignment and HKU3-6 for Lower RF and HGT

	**Position on alignment (aa)**	**Position on HKU3-6 (aa)**
**Lower RF**	520-680	894-1136
	770-870	1478-1635
	2930-3070	4279-4501
	4910-5080	6366-6544
**HGT**	360-1390	670-2585
	550-1610	924-2859
	680-1680	1136-2943
	700-1710	1163-2974
	2060-3090	3350-4521
	2130-3250	3420-4681

Table S3: Positions of windows on alignment and HKU3-6 for Lower RF and HGT

Table S4: Virus and Host Data

Strain	Virus genera	Host	Host type	Virus complete genome	Host_cytb
As6526	Beta	*Aselliscus stoliczkanus*	Insectivorous	KY417142	DQ888677
Bat coronavirus Cp/Yunnan2011	Beta	*Chaerephon plicatus*	Insectivorous	JX993988	ON640662
BtHp-BetaCoV/ZJ2013	Beta	*Hipposideros pratti*	Insectivorous	KF636752	OP894116
BtMf-AlphaCoV/AH2011	Alpha	*Miniopterus fuliginosus*	Insectivorous	KJ473795	AB085735
BtMf-AlphaCoV/JX2012	Alpha	*Miniopterus fuliginosus*	Insectivorous	KJ473796	AB085735
BtMf-AlphaCoV/GD2012	Alpha	*Miniopterus fuliginosus*	Insectivorous	KJ473797	AB085735
BtMf-AlphaCoV/HuB2013	Alpha	*Miniopterus fuliginosus*	Insectivorous	KJ473798	AB085735
BtMf-AlphaCoV/FJ2012	Alpha	*Miniopterus fuliginosus*	Insectivorous	KJ473799	AB085735
BtMf-AlphaCoV/HeN2013	Alpha	*Miniopterus fuliginosus*	Insectivorous	KJ473800	AB085735
AFCD62	Alpha	*Miniopterus magnater*	Insectivorous	EU420138	ON640726
AFCD307	Alpha	*Miniopterus pusillus*	Insectivorous	EU420137	MN366288
HKU8	Alpha	*Miniopterus pusillus*	Insectivorous	EU420139	MN366288
BtMr-AlphaCoV/SAX2011	Alpha	*Myotis ricketti*	Piscivorous	KJ473806	AB106608
BtPa-BetaCoV/GD2013	Beta	*Pipistrellus abramus*	Insectivorous	KJ473820	AB085739
HKU5-1	Beta	*Pipistrellus abramus*	Insectivorous	EF065509	AB085739
LYRa11	Beta	*Rhinolophus affinis*	Insectivorous	KF569996	KP972690
BtRs-BetaCoV/YN2018B	Beta	*Rhinolophus affinis*	Insectivorous	MK211376	KP972690
BtRs-BetaCoV/YN2018C	Beta	*Rhinolophus affinis*	Insectivorous	MK211377	KP972690
RaTG13	Beta	*Rhinolophus affinis*	Insectivorous	MN996532	KP972690
Bat_SL-CoV_BM48-31	Beta	*Rhinolophus blasii*	Insectivorous	GU190215	MZ936290
BM48-31/BGR/2008	Beta	*Rhinolophus blasii*	Insectivorous	NC_014470	MZ936290
BtRf-AlphaCoV/HuB2013	Alpha	*Rhinolophus ferrumequinum*	Insectivorous	KJ473807	AB085731
BtRf-AlphaCoV/YN2012	Alpha	*Rhinolophus ferrumequinum*	Insectivorous	KJ473808	AB085731
BtRf-BetaCoV/JL2012	Beta	*Rhinolophus ferrumequinum*	Insectivorous	KJ473811	AB085731
BtRf-BetaCoV/HeB2013	Beta	*Rhinolophus ferrumequinum*	Insectivorous	KJ473812	AB085731

Continued on next page

Table S4 – continued from previous page

**Strain**	**Virus genera**	**Host**	**Host type**	**Virus complete genome**	**Host_cytb**
BtRf-BetaCoV/SX2013	Beta	*Rhinolophus ferrumequinum*	Insectivorous	KJ473813	AB085731
Rm1	Beta	*Rhinolophus macrotis*	Insectivorous	DQ412043	KX261916
BtCoV/279/2005	Beta	*Rhinolophus macrotis*	Insectivorous	DQ648857	KX261916
Rp3	Beta	*Rhinolophus pearsoni*	Insectivorous	DQ071615	JX502551
Rp/Shaanxi2011	Beta	*Rhinolophus pusillus*	Insectivorous	JX993987	ON012504
F46	Beta	*Rhinolophus pusillus*	Insectivorous	KU973692	ON012504
HKU3-1	Beta	*Rhinolophus sinicus*	Insectivorous	DQ022305	HM134917
HKU3-2	Beta	*Rhinolophus sinicus*	Insectivorous	DQ084199	HM134917
HKU3-3	Beta	*Rhinolophus sinicus*	Insectivorous	DQ084200	HM134917
Rs672/2006	Beta	*Rhinolophus sinicus*	Insectivorous	FJ588686	HM134917
HKU3-4	Beta	*Rhinolophus sinicus*	Insectivorous	GQ153539	HM134917
HKU3-5	Beta	*Rhinolophus sinicus*	Insectivorous	GQ153540	HM134917
HKU3-6	Beta	*Rhinolophus sinicus*	Insectivorous	GQ153541	HM134917
HKU3-7	Beta	*Rhinolophus sinicus*	Insectivorous	GQ153542	HM134917
HKU3-8	Beta	*Rhinolophus sinicus*	Insectivorous	GQ153543	HM134917
HKU3-9	Beta	*Rhinolophus sinicus*	Insectivorous	GQ153544	HM134917
HKU3-10	Beta	*Rhinolophus sinicus*	Insectivorous	GQ153545	HM134917
HKU3-11	Beta	*Rhinolophus sinicus*	Insectivorous	GQ153546	HM134917
HKU3-12	Beta	*Rhinolophus sinicus*	Insectivorous	GQ153547	HM134917
HKU3-13	Beta	*Rhinolophus sinicus*	Insectivorous	GQ153548	HM134917
HKU9-1	Beta	*Rousettus leschenaultii*	Frugivorous	EF065509	EU076711
Ro-BatCoV-HKU9-1	Beta	*Rousettus leschenaultii*	Frugivorous	EF065509	EU076711
LYRa12	Beta	*Rousettus leschenaultii*	Frugivorous	KF569997	EU076711
BtRs-BetaCoV/YN2018A	Beta	*Rousettus leschenaultii*	Frugivorous	MK211374	EU076711
BtRs-BetaCoV/YN2018B	Beta	*Rousettus leschenaultii*	Frugivorous	MK211375	EU076711
BtRs-BetaCoV/YN2018C	Beta	*Rousettus leschenaultii*	Frugivorous	MK211376	EU076711

Continued on next page

Table S4 – continued from previous page

Strain	Virus genera	Host	Host type	Virus complete genome	Host_cytb
BtRa-BetaCoV/SX2013	Beta	*Rousettus leschenaultii*	Frugivorous	KJ473819	EU076711
BtCoV/273/2005	Beta	*Rousettus leschenaultii*	Frugivorous	DQ648856	EU076711
BtRs-BetaCoV/YN2013B	Beta	*Rousettus leschenaultii*	Frugivorous	KJ473818	EU076711
BtRl-BetaCoV/SAX2011	Beta	*Rousettus leschenaultii*	Frugivorous	KJ473817	EU076711
BtRl-BetaCoV/GD2013	Beta	*Rousettus leschenaultii*	Frugivorous	KJ473816	EU076711
BtRl-BetaCoV/HuB2013	Beta	*Rousettus leschenaultii*	Frugivorous	KJ473815	EU076711
BtRl-BetaCoV/SC2013	Beta	*Rousettus leschenaultii*	Frugivorous	KJ473814	EU076711
BtTp-BetaCoV/GX2012	Beta	*Tylonycteris pachypus*	Insectivorous	KJ473822	ON640722
HKU4-1	Beta	*Tylonycteris pachypus*	Insectivorous	EF065505	ON640722
BtVs-BetaCoV/SC2013	Beta	*Vespertilio superans*	Insectivorous	KJ473821	AB085738

References

[1] Agnarsson, I., Zambrana-Torrelio, C., Flores-Saldana, N. & May-Collado, L. A time-calibrated species-level phylogeny of bats (Chiroptera, Mammalia). *PLoS Currents.* **3** (2011)

[2] Bradley, R. & Baker, R. A test of the genetic species concept: cytochrome-b sequences and mammals. *Journal Of Mammalogy.* **82**, 960-973 (2001)

[3] Kocher, T., Thomas, W., Meyer, A., Edwards, S., Pääbo, S., Villablanca, F. & Wilson, A. Dynamics of mitochondrial DNA evolution in animals: amplification and sequencing with conserved primers.. *Proceedings Of The National Academy Of Sciences.* **86**, 6196-6200 (1989)

[4] Hu, B., Zeng, L., Yang, X., Ge, X., Zhang, W., Li, B., Xie, J., Shen, X., Zhang, Y., Wang, N. & Others Discovery of a rich gene pool of bat SARS-related coronaviruses provides new insights into the origin of SARS coronavirus. *PLoS Pathogens.* **13**, e1006698 (2017)

[5] Li, G., Liang, B., Wang, Y., Zhao, H., Helgen, K., Lin, L., Jones, G. & Zhang, S. Echolocation calls, diet, and phylogenetic relationships of Stoliczka's trident bat, Aselliscus stoliczkanus (Hipposideridae). *Journal Of Mammalogy.* **88**, 736-744 (2007)

[6] Yang, L., Wu, Z., Ren, X., Yang, F., He, G., Zhang, J., Dong, J., Sun, L., Zhu, Y., Du, J. & Others Novel SARS-like betacoronaviruses in bats, China, 2011. *Emerging Infectious Diseases.* **19**, 989 (2013)

[7] Wu, Z., Han, Y., Wang, Y., Liu, B., Zhao, L., Zhang, J., Su, H., Zhao, W., Liu, L., Bai, S. & Others A comprehensive survey of bat sarbecoviruses across China in relation to the origins of SARS-CoV and SARS-CoV-2. *National Science Review.* pp. nwac213 (2022)

[8] Wu, Z., Yang, L., Ren, X., Zhang, J., Yang, F., Zhang, S. & Jin, Q. ORF8-related genetic evidence for Chinese horseshoe bats as the source of human severe acute respiratory syndrome coronavirus. *The Journal Of Infectious Diseases.* **213**, 579-583 (2016)

[9] Du, J., Yang, L., Ren, X., Zhang, J., Dong, J., Sun, L., Zhu, Y., Yang, F., Zhang, S., Wu, Z. & Others Genetic diversity of coronaviruses in Miniopterus fuliginosus bats. *Science China Life Sciences.* **59** pp. 604-614 (2016)

[10] Sakai, T., Kikkawa, Y., Tsuchiya, K., Harada, M., Kanoe, M., Yoshiyuki, M. & Yonekawa, H. Molecular phylogeny of Japanese Rhinolophidae based on variations in the complete sequence of the mitochondrial cytochrome b gene. *Genes & Genetic Systems.* **78**, 179-189 (2003)

[11] Chu, D., Peiris, J., Chen, H., Guan, Y. & Poon, L. Genomic characterizations of bat coronaviruses (1A, 1B and HKU8) and evidence for co-infections in Miniopterus bats. *Journal Of General Virology.* **89**, 1282-1287 (2008)

[12] Wu, Z., Yang, L., Ren, X., He, G., Zhang, J., Yang, J., Qian, Z., Dong, J., Sun, L., Zhu, Y. & Others Deciphering the bat virome catalog to better understand the ecological diversity of bat viruses and the bat origin of emerging infectious diseases. *The ISME Journal.* **10**, 609-620 (2016)

[13] Kawai, K., Nikaido, M., Harada, M., Matsumura, S., Lin, L., Wu, Y., Hasegawa, M. & Okada, N. The status of the Japanese and East Asian bats of the genus Myotis (Vespertilionidae) based on mitochondrial sequences. *Molecular Phylogenetics And Evolution.* **28**, 297-307 (2003)

[14] Woo, P., Wang, M., Lau, S., Xu, H., Poon, R., Guo, R., Wong, B., Gao, K., Tsoi, H., Huang, Y. & Others Comparative analysis of twelve genomes of three novel group 2c and group 2d coronaviruses reveals unique group and subgroup features. *Journal Of Virology*. **81**, 1574-1585 (2007)

[15] He, B., Zhang, Y., Xu, L., Yang, W., Yang, F., Feng, Y., Xia, L., Zhou, J., Zhen, W., Feng, Y. & Others Identification of diverse alphacoronaviruses and genomic characterization of a novel severe acute respiratory syndrome-like coronavirus from bats in China. *Journal Of Virology*. **88**, 7070-7082 (2014)

[16] Zhou, P., Yang, X., Wang, X., Hu, B., Zhang, L., Zhang, W., Si, H., Zhu, Y., Li, B., Huang, C. & Others A pneumonia outbreak associated with a new coronavirus of probable bat origin. *Nature*. **579**, 270-273 (2020)

[17] Curran, M., Kopp, M., Ruedi, M. & Bayliss, J. A new species of horseshoe bat (Chiroptera: Rhinolophidae) from Mount Namuli, Mozambique. *Acta Chiropterologica*. **24**, 19-40 (2022)

[18] Drexler, J., Gloza-Rausch, F., Glende, J., Corman, V., Muth, D., Goettsche, M., Seebens, A., Niedrig, M., Pfefferle, S., Yordanov, S. & Others Genomic characterization of severe acute respiratory syndrome-related coronavirus in European bats and classification of coronaviruses based on partial RNA-dependent RNA polymerase gene sequences. *Journal Of Virology*. **84**, 11336-11349 (2010)

[19] Sun, K., Kimball, R., Liu, T., Wei, X., Jin, L., Jiang, T., Lin, A. & Feng, J. The complex evolutionary history of big-eared horseshoe bats (Rhinolophus macrotis complex): insights from genetic, morphological and acoustic data. *Scientific Reports*. **6**, 35417 (2016)

[20] Li, W., Shi, Z., Yu, M., Ren, W., Smith, C., Epstein, J., Wang, H., Crameri, G., Hu, Z., Zhang, H. & Others Bats are natural reservoirs of SARS-like coronaviruses. *Science*. **310**, 676-679 (2005)

[21] Wang, W., Tian, J., Chen, X., Hu, R., Lin, X., Pei, Y., Lv, J., Zheng, J., Dai, F., Song, Z. & Others Coronaviruses in wild animals sampled in and around Wuhan at the beginning of COVID-19 emergence. *Virus Evolution*. **8**, veac046 (2022)

[22] Lau, S., Woo, P., Li, K., Huang, Y., Tsoi, H., Wong, B., Wong, S., Leung, S., Chan, K. & Yuen, K. Severe acute respiratory syndrome coronavirus-like virus in Chinese horseshoe bats. *Proceedings Of The National Academy Of Sciences*. **102**, 14040-14045 (2005)

[23] Yuan, J., Hon, C., Li, Y., Wang, D., Xu, G., Zhang, H., Zhou, P., Poon, L., Lam, T., Leung, F. & Others Intraspecies diversity of SARS-like coronaviruses in Rhinolophus sinicus and its implications for the origin of SARS coronaviruses in humans. *Journal Of General Virology*. **91**, 1058-1062 (2010)

[24] Lau, S., Li, K., Huang, Y., Shek, C., Tse, H., Wang, M., Choi, G., Xu, H., Lam, C., Guo, R. & Others Ecoepidemiology and complete genome comparison of different strains of severe acute respiratory syndrome-related Rhinolophus bat coronavirus in China reveal bats as a reservoir for acute, self-limiting infection that allows recombination events. *Journal Of Virology*. **84**, 2808-2819 (2010)

[25] Ge, X., Li, J., Yang, X., Chmura, A., Zhu, G., Epstein, J., Mazet, J., Hu, B., Zhang, W., Peng, C. & Others Isolation and characterization of a bat SARS-like coronavirus that uses the ACE2 receptor. *Nature*. **503**, 535-538 (2013)

[26] Yang, X., Hu, B., Wang, B., Wang, M., Zhang, Q., Zhang, W., Wu, L., Ge, X., Zhang, Y., Daszak, P. & Others Isolation and characterization of a novel bat coronavirus closely related to the direct progenitor of severe acute respiratory syndrome coronavirus. *Journal Of Virology*. **90**, 3253-3256 (2016)
